# Oral exposure to lead for Japanese children and pregnant women, estimated using duplicate food portions and house dust analyses

**DOI:** 10.1186/s12199-019-0818-4

**Published:** 2019-12-05

**Authors:** Mayumi Ohtsu, Nathan Mise, Akihiko Ikegami, Atsuko Mizuno, Yayoi Kobayashi, Yoshihiko Nakagi, Keiko Nohara, Takahiko Yoshida, Fujio Kayama

**Affiliations:** 1 Department of Environmental and Preventive Medicine, Jichi Medical University, 3311-1, Yakushiji, Shimotsuke, Tochigi, 329-0498 Japan; 2 Department of Pharmacology, Jichi Medical University, 3311-1, Yakushiji, Shimotsuke, Tochigi, 329-0498 Japan; 3 Center for Health and Environmental Risk Research, National Institute for Environmental Studies, 16-2, Onogawa, Tsukuba, Ibaraki, 305-8506 Japan; 4 Department of Health Science, Asahikawa Medical University, Midorigaoka-higashi, Asahikawa, Hokkaido 078-8802 Japan

**Keywords:** Lead exposure, Duplicate food portion, House dust, Body burden analysis

## Abstract

**Background:**

Lead is a toxic metal abundant in the environment. Consumption of food contaminated at low levels of lead, especially by small children and pregnant women, raises a health concern.

**Methods:**

Duplicated food portions and drinking water were collected over 3 days from 88 children and 87 pregnant women in Shimotsuke, Tochigi, Japan. Participants were recruited in this study between January 2014 and October 2015. Dust was also collected from their homes. Lead concentrations were measured and consequent oral lead exposure levels were estimated for this population at high risk to environmental toxicants. Lead concentrations of peripheral and cord blood, taken from children and pregnant women, and were also analyzed.

**Results:**

Lead concentrations in food, drinking water, and house dust were low in general. Oral lead exposure to lead was higher for children (Mean ± SEM; 5.21 ± 0.30 μg/kg BW/week) than in pregnant women (1.47 ± 0.13 μg/kg BW/week). Food and house dust were main sources of lead contamination, but the contribution of house dust widely varied. Means ± SEM of peripheral and cord blood lead concentrations were 0.69 ± 0.04 μg/dL and 0.54 ± 0.05 μg/dL, respectively for pregnant women and 1.30 ± 0.07 μg/dL (peripheral only) in children. We detect no correlation between smoking situations and blood lead concentration in pregnant women.

**Conclusion:**

We conclude that oral lead exposure levels for Japanese children and pregnant women were generally low, with higher concentrations and exposure for children than for pregnant women. More efforts are necessary to clarify the sources of lead contamination and reduce lead exposure of the population at high risk even in Japan.

## Background

Lead is a highly toxic heavy metal ubiquitous in the environment, and found at trace levels in food, soil, water, house dust, and air [1]. Lead toxicity affects to almost all organs and organ systems, and it is known that the nervous system is the most vulnerable especially in developing fetuses and children [2, 3]. Developing fetuses and small children are more sensitive to environmental contaminants than adults [4, 5], and adverse effects due to exposure to lead of wide range concentration are concerning.

Prohibition of usage of leaded gasoline in the 1980s resulted in a remarkable decrease of environmental lead in Japan. It has been reported that concentrations of lead in blood of adults and children have been declining [6]. Acute exposure to lead is rare and sporadic at present in Japan; however, concerns about low-level chronic exposure to lead are rapidly growing. Until 2012 in the USA, children were considered as having a blood lead “level of concern” if the blood lead concentration was found to be higher than 10 μg/dL [7]. However, blood lead at concentrations below 5 μg/dL is a causal risk factor for diminished intellectual and academic abilities, higher rates of neurobehavioral disorders such as hyperactivity and attention deficits, and lower birth weight in children [8–12]. The US Centers for Disease Control and Prevention (CDC) stated that no safe threshold for blood lead level [13]. It has been suggested that no threshold of blood lead level for impact on children’s IQ cannot be found [9, 14], and it is now recommended that exposure to lead should be reduced to “as less as possible” [13].

Children are exposed to lead more often by hand-mouth behavior from time spent on dusty floors. Absorption of lead from the gastrointestinal tract is more efficient for children than that for adults [15]. In a pregnant woman, lead can cross the placenta to the fetus and can affect the developing prenatal fetus [16, 17]. Adverse effects of prenatal exposure to lead of developing fetus through the placenta by maternal circulatory system have already been reported [18–23]. Decreasing lead exposure for pregnant women and children is still needed; therefore, monitoring of lead exposure level of these populations at high risk is important [24].

Low-level environmental exposure to lead is associated with multiple sources and pathways [25]. In the indoor environment, oral ingestion of food and drinking water, as well as house dust, could be important sources of lead contaminations [6, 26]. Cigarette smoke is also known as an environmental source of lead [27–29]. In the course of our exposure monitoring project, named “PbAs study,”we examined oral exposure to inorganic arsenic and lead through food, drinking water, and house dust and monitored the lead concentrations in peripheral blood of children and pregnant women and cord blood of fetuses as biomarkers of short-term lead exposure [30–32].

Small children and pregnant women are populations at high risk when exposed to lead and it is necessary to keep evaluating their current exposure status. To examine the oral exposure to lead in children, fetuses, and pregnant women in Japan, drinking water and duplicated portions of food for 3 days were collected, and concentration of lead in these materials were measured. There were few studies analyzing food duplicated portions of three consecutive days (72 h) in Japan; so far, 24-h duplicate food portion was usually used as a typical food [33, 34]. Seven-day house dust collected by a vacuum cleaner was also collected to measure concentration of lead. Weekly oral exposure to lead, on a body weight basis, was estimated as the sum of lead ingestion through house dust, drinking water, and food. Lead concentrations in the peripheral blood of pregnant women and children and umbilical cord blood were also analyzed. Using these samples, we could evaluate the short-term exposure to lead of pregnant, prenatal, and childhood periods at the same time. Generally, oral exposure levels for pregnant women and children were low, as reported earlier [2, 6, 33]. We found that exposure levels and blood lead concentrations for children were higher than those of pregnant women and that the contribution from house dust to the oral exposure levels of children were high. We could not detect any relationships between lead blood concentrations and active or passive smoking situations.

## Methods

### Subjects

This study was a part of our project studying dietary exposure assessment to Pb (lead) and inorganic As (arsenic), named the “PbAs study.” Details of arsenic exposure in the same subjects have been published elsewhere [32]. Pregnant women who already had young children (1–3.5 years) were recruited for our duplicated diet study from one local hospital and five clinics after explanation of the project by trained nurses or obstetricians. Participants were recruited in this study between January 2014 and October 2015. Pregnant women in first to second trimesters and already having a child of 1 to 3.5 years of age (*n* = 89 in Shimotsuke) participated in our study. We obtained full samples and data from 86 pregnant women and 87 children in Shimotsuke. To increase the number of participants and to compare the regional differences of lead exposure levels, we tried to recruited pairs of pregnant women and children in Asahikawa, Hokkaido at the same time. However, due to small numbers of birth in Asahikawa area, we could not obtain enough number of participants. Children of 1 to 3.5 years old were enrolled for lead exposure analysis in children in Asahikawa (*n* = 14). We compared regional differences of lead exposure levels between Shimotsuke and Asahikawa and did not get statistically significant differences (Additional file 1: Table S1). Due to inconsistency of sampling conditions, all Asahikawa data has not been included into the main analyses. The participants also filled a self-administered questionnaire on family and health conditions including smoking and passive smoking condition of pregnant women at time of enrolment. Brief familial conditions were summarized in Table 1, modified from Mise et al. [32].

**Table 1:** Summary of the familial condition of participating pregnant mothers and children (modified from Mise et al. (2019))

	*n*	Median (range) or incidence
Pregnant women		
Age (years)	89	32.2 (22.0–43.0)
Body weight (kg) in pre-pregnancy	89	59.6 (42.0–97.0)
Collected food duplicate portion	88	
Collected drinking water	63	
Children		
Age (years)	89	2.4 (1.3–3.4)
Sex		
Boy	54	60.7%
Girl	35	39.3%
Weight at the time of collecting of food duplicates (kg)	88	12.0 (7.6–17.0)
Collected food duplicate portion	88	
Collected drinking water	56	

### Collection of duplicated diet portions and drinking water

All protocols of collecting duplicate food portions have been previously described [32]. Briefly, the participants were asked to collect all foods consumed over 3 days, including two weekdays and one weekend day. The duplicate diet portions included beverages, supplements, and snacks. Drinking water taken from the domestic water supply or purchased mineral water was collected as drinking water, but beverages, such as tea, coffee, and soft drinks, were collected as food items. The participants who did not provide drinking water were considered as taking all water from beverages those included in the food duplicated portions. The contribution of drinking water of lead intake was calculated as “zero” in such cases. Collected duplicate diet portions and drinking water samples were sent to the laboratory by a commercial delivery service. All samples were weighed on arrival.

### Collection of peripheral blood of pregnant women and children and cord blood

Peripheral blood samples from children and pregnant women were collected by the physicians when the subjects visited our laboratory. When the visits of the pregnant women were scheduled after their delivery, the blood specimen collection was carried out in the hospital or clinic during their hospital stay. Local anesthetic cream, 5% EMLA Cream (Astra Zeneaca, Cambridge, UK), was used to decrease pain from piercing for children. Cord blood samples were collected by the obstetricians in a blood sampling bag (TERUMO, Tokyo, Japan) at the parturition in the clinics. The collected blood samples were stored at − 80 °C until analysis. The collection times of duplicate food portions and peripheral bloods were not at the same period because of the participants’ convenience.

### Collection and preparation of house dust

Routine cleaning using a bagless vacuum cleaner was carried out to collect the dust from dry living areas in a subject’s house during the period of collection of duplicate food portions. Before the start of collecting house dust, the whole living area of the participants house were cleaned by participants, and all the dust was removed from the cleaner to avoid collecting house dust that remained for a long period in the house. The house dust collected during the 7 days was estimated as normal house dust and was used to validate the lead concentration. Samples were collected from 105 houses in total, 88 houses in Shimotsuke and 17 houses in Asahikawa, and Shimotsuke samples were used in the analyses as described above. Each vacuum dust sample was passed through a sieve with an opening size of 100 μm (Tokyo Screen, Tokyo, Japan) according to the instruction of the National Institute of Standards and Technology (NIST) Standard Reference Material (SRM) 2583 (Trace Elements in Indoor Dust). Hairs and fibrous materials were manually removed from the sample during the preparation. The house dust was then dried at 60 °C overnight in a drying oven. To avoid cross-contamination, house dust samples were kept in separate plastic bags in a cool and dry environment, away from sunlight and fumes.

### Sample preparations and lead measurement by ICP-MS

Detailed preparation protocols for foods, drinking water, and blood for quantification by ICP-MS have already been described [30]. All 3-day duplicate portions were mixed and homogenized thoroughly before metal extraction. Concentration in the food mixture was considered as averaged lead concentration in daily foods. The lead concentrations in blood, food, and drinking water were determined using ICP-MS. The ICP-MS Agilent 7500cx (Agilent Technologies Japan, Tokyo, Japan) in The National Institute for Environmental Studies (NIES), Japan was employed to measure the lead concentrations. The measurement for lead was carried out by the calibration curve method using a lead standard solution (Wako Pure Chemical Industries, Ltd., Osaka, Japan) and a thallium standard solution (Wako Pure Chemical Industries, Ltd., Osaka, Japan) for an internal standard. The lower limit detection of lead was 0.001 ng/mL (ppb). The test for quality control was performed by using commercial reference samples: National Institute of Standards and Technology (NIST) Standard Reference Material (SRM) 995c, Toxic Metals in Caprine Blood (NIST, Gaithersburg, MD, USA) for blood analysis; National Metrology Institute of Japan (NMIJ, Tsukuba, Japan) Certificated Reference Materials (CRM) 7202-b, Trace Elements in River Water (Elevated Level) (NMIJ, Tsukuba, Japan) for water analysis. The recovery of lead for the blood and water analysis methods was 95.7% and 92.9%, respectively [30].

### EDXRF analysis of house dust

Energy dispersive X-ray fluorescence spectrometry (EDXRF) was performed to determine heavy metal concentrations in house dust samples. EDXRF analysis was conducted using the Industrial Technology Center of Tochigi Prefecture JSX-3100RII element analyzer (JEOL, Tokyo, Japan). House dust sample was placed in a specific plastic cup with thin film supports of PROLENE 4.0 μm (Chemplex Industries, Florida, USA). The samples were analyzed for 240 s (live time) using X-ray lamp voltage of 50 kV, auto lamp current, 7 mm collimator, and Pb filter. The measurement of lead was carried out using the calibration curve method equipped in the instrument and the region of interest for Pb setting 12.4–12.9 keV of Lβ1. Samples for calibration were prepared by mixing cellulose, 100 μm sieved powder (Nacalai Tesque, Kyoto, Japan), and NIST SRM 2583. The lower limit of detection (LLD) for lead was 2.5 μg/g. Lead concentrations less than the LLD were assigned the half value of LLD. Quality control test was performed using NIST SRM 2583 (trace elements in indoor dust). The recovery of lead for the analysis method was 99.4%.

### Calculation for intake of Pb from food, water, and house dust

#### Daily intake

Daily Pb intake from food [μg/day] = concentration of Pb [μg/g] × daily food consumption [g/day] = [A]

Daily Pb intake from water [μg/day] = concentration of Pb [μg/g] × daily drinking water consumption [g/day] = [B]

The ingestion rates of house dust were estimated as 60 mg/day for a child (age: 1–6 years) and as 30 mg/day for an adult according to the US Environmental Protection Agency (EPA) Exposure Factors Handbook 2011 Edition (EPA) [35].

Takagi and Yoshinaga [36] reported that 50% and maximum values of ingestion rate for Japanese children were 25 and 200 mg/day, respectively. In addition, according to a recent study, the ingestion rate of house dust was estimated as 100 mg/day (general population upper percentile, US EPA, 2011) for Japanese children [35]. We therefore selected the ingestion rate of house dust 60 mg/day (general population central tendency, US EPA, 2011) to prevent an overestimation of contribution of house dust on diet (food + water).

Daily Pb intake of pregnant woman from house dust [μg/day] = concentration of Pb [μg/g] × 0.03 [g/day] = [C]

Daily Pb intake of child from house dust [μg/day] = concentration of Pb [μg/g] × 0.06 [g/day] = [D]

### Weekly in take per body weight

Weekly Pb intake from food [μg/kg BW/week] = ([A] [μg/day] × 7 [days]) / body weight at sampling time[kg] = [E]

Weekly Pb intake from drinking water [μg/kg BW/week] = ([B] [μg/day] × 7 [days]) / body weight at sampling time [kg] = [F]

Weekly Pb intake from house dust [μg/kg BW/week] = ([C] for pregnant woman or [D] for child [μg/day] × 7 [days]) / body weight at sampling time [kg] = [G]

Total weekly intake of Pb [μg/kg BW/week] = [E] + [F] + [G]

### Smoking situation

Smoking situation of the participants were extracted from the self-administered questionnaire. The participants who maintained their cigarette habitation during pregnancy (*n* = 3) and who stopped smoking at the time when they noticed their gestation (*n* = 7) were regarded as “Active smoker (*n* = 10)”. The participants who described in the questionnaire that they were exposed to secondhand smoke at home and/or at work were classified in the “Passive smokers (*n* = 26)”.

### Statistical analyses

All statistical analyses were conducted using R ver. 3.5.1 (https://www.r-project.org/). All results were indicated as arithmetic Mean ± standard error of mean (SEM). All the data of weekly lead intake per body weight and the lead concentrations in blood samples were assessed for normality using the Shapiro-Wilk test. Distributions of all of these data were skewed (data not shown, *p* < 0.05). Non-parametric Wilcoxon-Mann-Whitney (WMW) test was used for bivariate comparisons of lead exposure levels. Multivariate comparison of the blood lead concentration in peripheral blood of pregnant women and children and cord blood was carried out using non-parametric Steel-Dwass test utilizing a script running on the R program obtained from a web site (http://aoki2.si.gunma-u.ac.jp/R/Steel-Dwass.html). Spearman’s rank correlation was utilized in all correlation analyses. A *p* value of < 0.05 was considered to be statistically significant in all statistical analyses.

## Results

### Oral Pb exposure of Japanese pregnant women and children

**Fig. 1:**
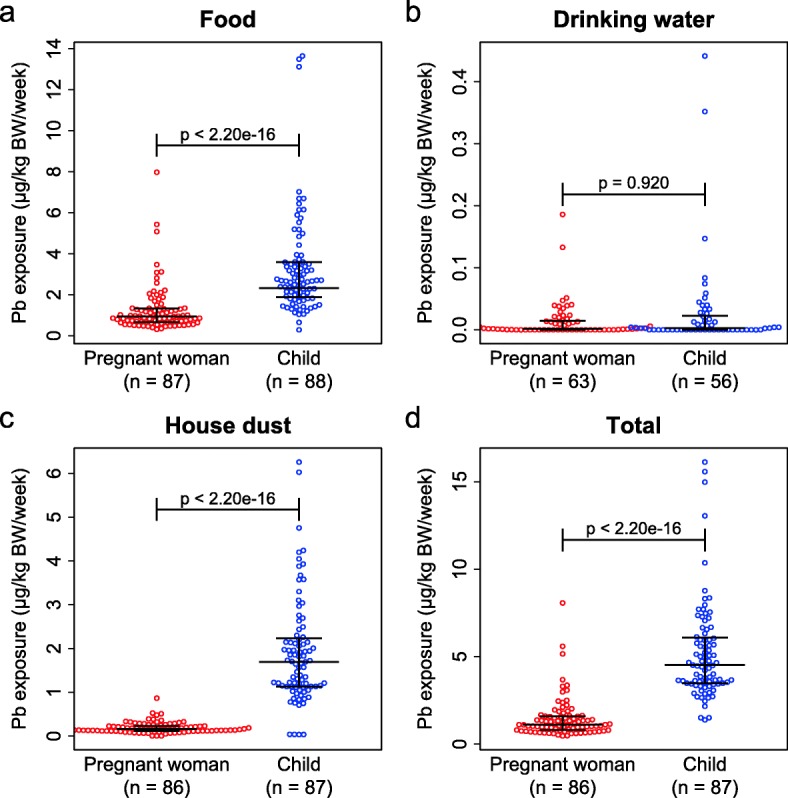
Lead intake per body weight per week from food, drinking water, and house dust. Beeswarm plot of oral exposure of Pb adjusted to the body weight per week (μg/kg BW/week) from food samples (**a**), drinking water samples (**b**), house dust samples (**c**), and total lead exposure (**d**). Medians (longer horizontal bars in the middle) and 1st and 3rd quartiles (lower and higher horizontal bars, respectively) were indicated in the graphs. The *p* values were indicated in the graphs (WMW test)

We analyzed lead concentrations in the duplicate portions of food, drinking water using ICP-MS, and in house dust using EDXRF (Additional file 2: Table S2). Figure 1 summarized data of weekly lead intake per body weight of pregnant women and children from food, drinking water, house dust, and in total. Lead exposure through drinking water was very low compared to other sources, and no statistical difference was found between pregnant women and children (Mean ± SEM; 0.01 ± 0.00 μg/kg BW/week and 0.03 ± 0.01 μg/kg BW/week, respectively; *p* = 0.920, WMW test). On the other hand, we found that weekly lead exposure levels per body weight of children from food (3.28 ± 0.26 μg/kg BW/ week) and house dust (1.90 ± 0.13 μg/kg BW/week) were significantly higher than those of pregnant women (food 1.23 ± 0.10 μg/kg BW/week; house dust 0.19 ± 0.01 μg/kg BW/week; both *p* values < 2.20 × 10^−16^, WMW test). Figure 1d shows a beeswarm plot of total oral lead exposure of pregnant women (1.47 ± 0.13 μg/kg BW/week) and children (5.21 ± 0.30 μg/kg BW/week). The level of total exposure was higher in children than in pregnant women (WMW test, *p* < 2.2 × 10^−16^). Table 2 summarized the contribution of the house dust in oral lead exposure, and the house dust contribution was higher in children (mean 38.4%, range 0.6 to 81.3) than in pregnant women (mean 16.1%, range 0.35 to 55.5). The age of the children analyzed in this study ranged 1 to 3.5 years old. The food items and cooking methods are rapidly changing in this period, and food consumption mass increases accompanying growth of children. We analyzed difference of lead intake according to age of children to evaluate the differences of exposure to lead accompanying growth but could not detect significant differences of weekly lead intake per body weight from food and drinking water (Additional file 3: Table S3).

**Table 2:** Contribution of house dust in oral lead exposure (%)

	House dust contribution (%)	
	Children	Pregnant women
Minimum	0.6	0.3
1st quartile	26.9	9.4
Median	37.5	14.0
Mean	38.4	16.1
3rd quartile	48.3	21.9
Maximum	81.3	55.5

### Blood lead concentrations in pregnant women, cord blood, and children

**Fig. 2:**
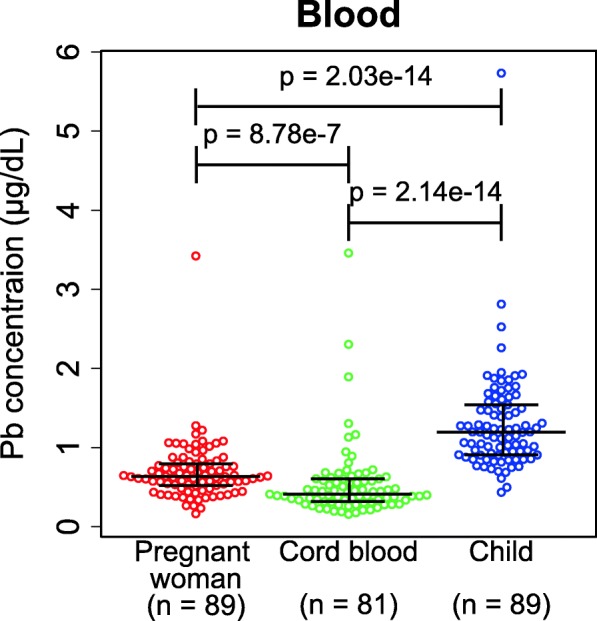
Lead concentration in blood of pregnant women, cord blood, and children. Lead concentration in peripheral blood of pregnant women and children and cord blood collected when the pregnant women’s delivery. Medians (longer horizontal lines) and 1st and 3rd quartiles (lower and higher horizontal lines, respectively) were indicated in the graph. Lead exposure of pregnant women and children were compared using with Steel-Dwass test. The *p* values were indicated in the graph

Figure 2 shows the lead concentrations in peripheral blood of pregnant women(Mean ± SEM; 0.69 ± 0.04 μg/dL), children (1.30 ± 0.07 μg/dL), and cord blood (0.54 ± 0.05 μg/dL). The lead concentration of cord blood was significantly lower than that of peripheral blood of pregnant women, and the lead concentration of peripheral blood of children was higher than that of the other samples (Steel-Dwass test, *p* values indicated in the plot). The children that participated in this study are in the rapidly growing period; their metal metabolism would be possibly changing during this period. We compared lead blood concentrations of children dividing into three different ages; however, we could not detect significant difference of blood lead concentrations (Additional file 3: Table S3).

### Correlation between blood lead concentrations and total oral lead exposure

We compared the total lead exposure level estimated from above analyses and blood lead concentrations of pregnant women, cord blood, and children. We found weak correlation between blood lead concentration in children and lead exposure from house dust (*p* = 0.029, *ρ* = 0.234) but failed to detect correlations of statistical significance in other combinations (Table 3).

**Table 3:** Correlations between blood lead concentrations and oral lead exposure level from various sources

		House dust	Water	Food	Total
Pregnant women	*p* value*	0.970	0.338	0.193	0.285
	*ρ*	0.004	0.123	0.141	0.116
Cord blood	*p* value*	0.579	0.969	0.127	0.090
	*ρ*	0.173	0.005	0.173	0.193
Children	*p* value*	0.029	0.387	0.249	0.118
	*ρ*	0.234	0.118	0.124	0.169

*p values obtained by Spearman’s rank correlation *ρ*

### Effects of smoking experience and passive smoking to blood lead concentration

**Fig. 3:**
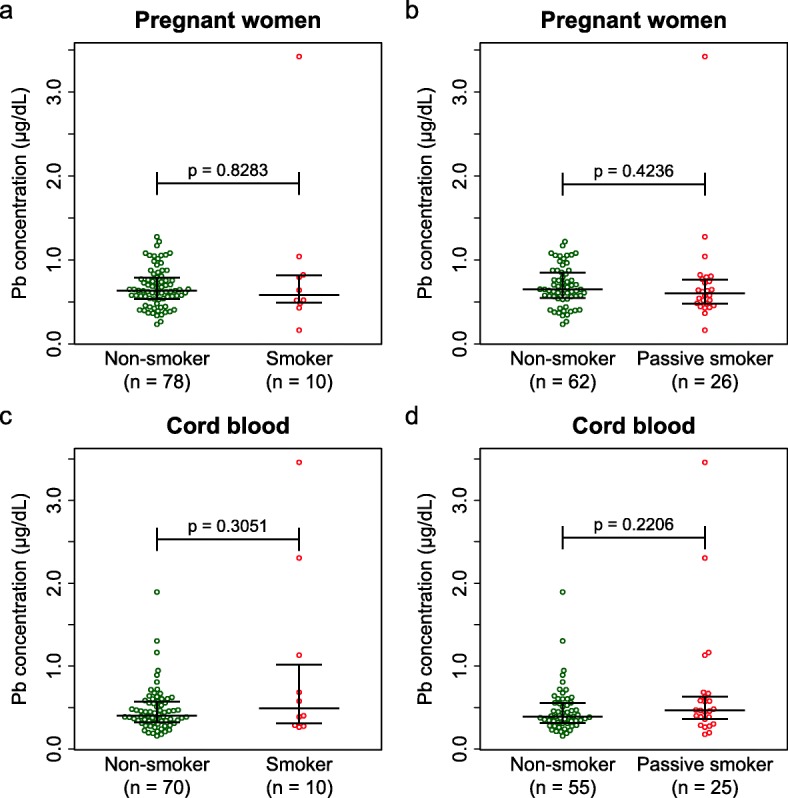
Smoking experience and passive smoking. Comparison of Pb concentrations in blood of pregnant women (**a**, **b**), cord blood (**c**, **d**) between non-smokers and smoking experienced (**a**, **c**), or between non-smokers and passive smokers (**b**, **d**). There was no statistical significance in all comparisons (WMW test)

We investigated the relationship between smoking and blood lead concentrations in pregnant women and cord blood, because cigarette smoking and secondhand smoking are possible sources of lead contamination [37]. Figure 3 shows the results of comparisons of lead concentrations in blood samples. We failed to detect any statistical significance among these comparisons (WMW test, *p* > 0.05).

## Discussion

In this study, we measured lead concentrations in food, drinking water, and house dust to estimate oral exposure to lead for children and pregnant women in Japan. The levels of oral lead exposure were not very high, as reported earlier [2, 6], and the data indicated that the food and house dust are the major sources of lead contamination for these populations at high risk in Japan. The blood lead level is taken as a biomarker of short-term lead exposure. The estimated level of lead exposure for children was higher than that for pregnant women, and the lead concentration in children’s blood was the highest among the samples. The levels of lead exposure for children and pregnant women were low with respect to other countries, and the effects of smoking and passive smoking were not significant.

Among the three different types of samples we collected in this study, drinking water was the lowest contributor to lead exposure. The maximum concentration of lead in drinking water in this study was 4.86 ng/mL, consistent with the results reported in [6]. Contamination of drinking water by lead usually occurs due to usage of lead pipelines, which have already been replaced in Japan [6, 33]. It has been pointed out that lead contamination in water during the analytical procedure could likely occur due to its low concentration and materials of containers [6]; therefore, we could not exclude the possibility that our results present in this study includes overestimations. In any case, lead exposure through consumption of the public water supply and bottled water could be considered to be of a lesser hygienic concern in Japan than has been previously reported [38].

Watanabe et al. reported dietary lead intake of children in Miyagi, Japan [34]. Their analysis was based on 24-h food duplicate portions of 296 children in Japan 2013, and the GM was 0.84 μg/kg BW/week. GM of lead exposure level of food of children in this study was 2.69 μg/kg BW/week. This difference might reflect regional difference of lead distribution. Aung et al. [33] has reported that mean of dietary intake of lead of Tokyo metropolitan area was 4.79 μg/day that was comparable to our results (mean of dietary lead intake per day; 5.59 μg/day).

The major sources of oral exposure to lead for children and pregnant women were found to be food and house dust. The contribution of house dust to the lead exposure was prominent, especially for children but varied among samples (range 0.4 to 30.7% in pregnant women and 0.6 to 81.3% in children). The geometric mean of concentration of lead in house dust in this study was 40.93 μg/g (range 1.20–234.00), which was lower than the results of Yoshinaga (2012) (geomean 97.8 μg/g, range 18.0–1800) [6]. We do not exclude the possibility of geographical differences in lead distribution or a further decreasing of lead concentration in Japan environment since the earlier study. In addition, Yoshinaga (2012) also compared the concentrations of lead in house dust in several countries. Lead concentration in house dust in this study (geomean: 40.93 μg/g, median: 46.00 μg/g) was lower than in the countries listed in the paper, i.e., UK (geomean: 150 μg/g), Australia (geomean: 85.2 μg/g), Canada (geomean: 233 μg/g), Hong Kong (Median: 157 μg/g), and Poland (geomean: 131–194 μg/g) [6]. On the other hand, food duplicate studies analyzing the levels of lead exposure through food have already been published by groups of several countries. Although direct comparison is difficult because of differences of methodology, very low lead intake through food has been reported in the duplicate food portion studies of children in South Korea (Median: 3.30 μg/kg BW/week, age 0 to 6 years) [39] and Brazil (Mean: 1.26 μg/kg BW/week, age 1 to 4 years) [40]. The results obtained in our study (Mean: 3.28 μg/kg BW/week, Median 2.67 μg/kg BW/week, age 1 to 3.5 years) was comparable to the results of these countries and were several times lower than those of China (Median: 15.66 μg/kg BW/week [41] or 11.55 μg/kg BW/week [42], age 1.9 to 7 years) and Poland (Mean: 11.57 μg/kg BW/week, age 4 to 6 years) [43]. Lead concentration in drinking water was quite low in Japan, as mentioned above. These results suggested that oral exposure to lead for Japanese children was maintained in lower level.

Yoshinaga et al. (2012) compared blood lead concentration of Japanese children (geomean: 1.07 μg/dL) and other countries and reported the lower blood lead concentration in Japanese children comparable level to that of Canada, USA, Germany, and Korea [2]. Higher lead concentrations of child blood were found in South Africa (Mean: 6.4 μg/dL), China (Mean: 8.07 μg/dL), and India (Mean: 8.36 μg/dL) [2]. Arithmetic and geometric mean of the blood lead concentration in our study was 1.30 and 1.20 μg/dL, respectively, and was similar level to that of the results of Japanese children presented in [2]. Recently, Nakayama et al. [44] reported using multiple birth cohort data that blood lead concentration of children in several countries, especially in Japan, is kept in very low level. However, it is difficult to directly compare the results of international data because of the differences in methodology, variation of sampling strategies, and some analytical uncertainty.

In this study, food, drinking water, and house dust were considered as the sources of oral exposure to lead. In general, airborne particles could also be a lead source; however, we did not collect any air samples. As reported, the concentration of lead in the atmosphere has rapidly decreased following the prohibition of leaded gasoline in 1980s and is still trending to decline [6]. It is well known that cigarette smoke contains lead and has been suggested that exposure to cigarette smoke increases the concentration of lead in blood [21]. We compared blood lead concentrations of smokers and those participants who reported being exposed to passive smoke with those from non-smokers. The group of smokers contained persons who stopped smoking after knowing their own pregnancy as lead retained in the bones and might elevate blood lead concentrations for a while. Contrary to an expectation that lead blood concentration is higher in smoker group, there were no statistical difference between smokers and non-smokers or between passive smokers and non-smokers. The number of smokers was too small to detect the effects of cigarette smoke to the lead blood concentration, and we did not collect any information concerning other possible confounding factors. Although we did not obtain the information concerning passive smoking situations of children that participated in this study, the notion of isolating children from cigarette smoke is well accepted these days in Japan. For example, Tokyo metropolitan government has enacted the regulations protecting people from passive smoking indoors in April 2018 [45], and we sometimes hear that smokers in family are forced to smoke outside of residences to isolate children from secondhand smoke. It has been reported that blood lead concentrations of Japanese children were affected by passive smoking, but the effect of cigarette smoke might be small [46]. Although it has not been clarified as the predominant source of lead in Japan, soil is estimated to be one of the sources [47]. We have not analyzed lead concentration of soil surrounding their residence or sandpits of favorite park of the children, and it might also be non-negligible to clarify the lead source [33, 48]. Direct ingestion of soil particles can take place in the playground especially for children participating in this study aged from 1 to 3.5 years old. Toys of children are also possible candidates of lead sources surrounding children, and it has been suggested that lead in paint on toys poses a health risk to children [49–52]. Although we have not found children having very high blood lead concentration in this study, sporadic lead exposure can happen through highly contaminated materials such as imported toys [52]. It would be interesting to analyze correlation between blood lead concentrations and the lead concentrations of soil and toys surrounding children and pregnant women in the participants of this study.

In this study, duplicate food portion, drinking water, and house dust were collected at the same time, but blood sample collection could not be carried out at the same time due to the participants’ convenience. Concerning cord blood, it was possible to collect it only at the time of the delivery. Therefore, it was not possible to exclude the time difference of collection time between blood and other items. This might be one of possible reasons for lack of the correlation between blood lead concentration and oral exposure to lead evaluated in this study. This was one of the limitations of this study to interpret the results. The sample size of our study is too small to be representative of the whole Japan, and distribution of lead in Japan is geographically varied as reported in [6]. It has been reported that blood lead concentration is affected by various parameters such as blood pressure and alcohol consumption those related to the bone metabolism [53], and statistical analysis adjusting confounding factors would be necessary to know the cause of variations of blood lead concentration.

## Conclusions

In this study, we have investigated the oral exposure level of pregnant women and children in Japan. Although the contribution rates were varied among the samples, the highest contribution was observed in house dust. However, the concentrations in house dust were found to be maintained in relatively lower level. Blood lead concentrations in children were higher than in pregnant women and cord blood but was maintained at very low level compared to other countries as reported in Yoshinaga et al. [2]. These results indicated that the level of the oral exposure to lead of pregnant women and children in Japan were very low.

## Supplementary information

**Additional file 1.** Table S1 Regional differences of lead exposure level.

**Additional file 2.** Table S2 Lead concentrations in collected samples.

**Additional file 3.** Table S3 differences of Pb intake and blood lead concentrations in children.

## Data Availability

Data will be made available upon request to the corresponding author, NM (nmise@jichi.ac.jp).
